# Annual acknowledgement of manuscript reviewers

**DOI:** 10.1186/s40249-015-0047-z

**Published:** 2015-04-21

**Authors:** Xiao-Nong Zhou

**Affiliations:** National Institute of psitic Diseases, Shanghai, China CDC

## Abstract

The editors of *Infectious Diseases of Poverty* would like to thank all our reviewers who have contributed to the journal in Volume 3 (2014).

**Noorizan Abd. Aziz**

Malaysia

**Kwame Adjei**

Ghana

**Pascale Allotey**

Malaysia

**Augustina Annan**

Ghana

**Margaret Cameron Baker**

United States of America

**Yan Bao**

China

**Edi Basuno**

Indonesia

**Tefera Belachew**

Ethiopia

**Robert Bergquist**

Sweden

**Franziska Bieri**

Australia

**David Blair**

Australia

**Ronald Blanton**

United States of America

**Boakye Boatin**

Ghana

**Andrea Bosman**

Switzerland

**Cameron Bowie**

Malawi

**Graham Brown**

Australia

**Colin Butler**

Australia

**C.P. Ramachandran**

Malaysia

**Le Canh**

Viet Nam

**Miguela Ayala Caniza**

United States of America

**Kwang Poo Chang**

United States of America

**Arvind Chavali**

United States of America

**Jun-hu Chen**

China

**Sadegh Chinikar**

Iran

**Lester Chitsulo**

Switzerland

**Ulisses Confalonieri**

Brazil

**Maria Milagros Cortez Alcobedes**

Venezuela

**Aditya Dash**

India

**Denis Daumerie**

Switzerland

**Bourgeois Denis**

France

**Meghnath Dhimal**

Nepal

**Emilio Dirlikov**

Canada

**Hui-Fen Dong**

China

**Bolaji Egbewale**

Nigeria

**Uwem Ekpo**

Nigeria

**William Ellis**

United Kingdom

**Agola Eric**

Kenya

**Hildegund Ertl**

United States of America

**Ashley Fox**

United States of America

**Qing Fu**

China

**Christian Gericke**

Australia

**Nancy Gibson**

Canada

**Travis Glenn**

United States of America

**Consuelo Gonzalez-Suarez**

Philippines

**Warwick Grant**

Australia

**Mohan Gupte**

India

**James Harrison**

United Kingdom

**Fang Huang**

China

**Nguyen Hung**

Viet Nam

**Jean Hunleth**

United States of America

**Michael Hust**

Germany

**Minjun Ji**

China

**Marie-Laure Joly-Guillou**

France

**Mamadou Kaba**

South Africa

**Unni Krishnan Karunakara**

United States of America

**Eili Klein**

United States of America

**Merav Kliner**

United Kingdom

**Alison Krentel**

Canada

**Navasona Krishnan**

United States of America

**Yashwant Kumar**

India

**Kang Yiu Lai**

Hong Kong

**Lydia Leonardo**

Philippines

**Xin-Xu Li**

China

**Shi-zhu Li**

China

**Lu Liu**

China

**Knut Lönnroth**

Switzerland

**Shan Lv**

China

**Zhi-yue Lv**

China

**Pascal Magnussen**

Denmark

**Vivi Maketa Tevuzula**

Congo, Democratic Republic

**Kohei Makita**

United Kingdom

**Hein Mallee**

Japan

**Gino C. Matibag**

Philippines

**Rose McGready**

Thailand

**Rajeev Mehla**

India

**Gokhan Metan**

Turkey

**Antonio Montresor**

Switzerland

**Filbert Mpogoro**

Tanzania

**Samson Mukaratirwa**

South Africa

**Afework Mulugeta**

Ethiopia

**Erick Muok**

Kenya

**Manoj Murhekar**

India

**Diakite Nana Rose**

Cote d'Ivoire

**Mercy Nassali**

Bangladesh

**Jintana Ngamvithayapong-Yanai**

Thailand

**Abel Ntambue**

Congo, Democratic Republic

**John Nuckols**

United States of America

**Wilson Odero**

Kenya

**Hiroshi Ohmae**

Japan

**Olaniyi Olayinka**

United States of America

**Wellington Oyibo**

Nigeria

**Mariya Pakharukova**

Russian Federation

**Kevin Palmer**

United States of America

**Parviz Parvizi**

Iran

**Mohan Paudel**

Nepal

**Guey Chuen Perng**

Taiwan

**Eric Pevzner**

United States of America

**Renaud Piarroux**

France

**Li-ping Duan**

China

**Rebecca Pobocik**

United States of America

**Xiao-peng Qi**

China

**Men-Bao Qian**

China

**Jan Remme**

France

**Carsten Richter**

China

**Mohammad Bagher Rokni**

Iran

**Bishnupada Roy**

India

**Charles Rupprecht**

United States of America

**Moussa Sacko**

Mali

**Rehana Salam**

Pakistan

**Fadjar Satrija**

Indonesia

**Somphou Sayasone**

Laos

**Leonhard Schnittger**

Argentina

**Babar Tasneem Shaikh**

Pakistan

**Bhavna Shroff**

United States of America

**Johannes Sommerfeld**

Switzerland

**Ireneous N. Soyiri**

Malaysia

**Maja Stanojevic**

Serbia

**Jennifer Steele**

United States of America

**Peter Steinmann**

Switzerland

**Hiko Tamashiro**

Japan

**Ernest Tambo**

South Africa

**Anupama Tantri**

United States of America

**Li-guang Tian**

China

**Kingsley Nnanna Ukwaja**

Nigeria

**Fred Unger**

Germany

**Thomas Unnasch**

United States of America

**Raman Velayudhan**

Switzerland

**Carol Vlassoff**

Canada

**Wei Wang**

China

**Yong Wang**

China

**Andrew Ward**

United States of America

**Ting-huan Wen**

China

**Bruce Wilcox**

Thailand

**Maria Digna Winda Widyastuti**

Indonesia

**Viroj Wiwanitkit**

Thailand

**Zhong-Dao Wu**

China

**Shang Xia**

China

**Zheng Xie**

China

**Li-li Xu**

China

**Jing Xu**

China

**Kun Yang**

China

**Pin Yang**

China

**Jian-Hai Yin**

China

**Tai-Soon Yong**

Korea South

**Feng-Yi Qu**

China

**Shun-xian Zhang**

China

**Zai-xing Zhang**

Solomon Islands

**Xiao-Nong Zhou**

China

**Xia Zhou**

China

**Yi-biao Zhou**

China

**Shui-sen Zhou**

China

**Huai-min Zhu**

China

**Jakob Zinsstag**

Switzerland

**Rutendo B.L. Zinyama-Gutsire**

Zimbabwe

**Denis Zofou**

Cameroon

